# Hair Washing Formulations from *Aloe elegans* Todaro Gel: The Potential for Making Hair Shampoo

**DOI:** 10.1155/2020/8835120

**Published:** 2020-08-29

**Authors:** Desta Berhe Sbhatu, Goitom Gebreyohannes Berhe, Abadi Gebreyesus Hndeya, Asmael Abdu, Afework Mulugeta, Haftom Baraki Abraha, Micheale Yifter Weldemichael, Hailekiros Tadesse Tekle, Haftay Abadi Gebru, Molla Gereme Taye, Haileselassie Gebremeskel Kidanemariam

**Affiliations:** ^1^Mekelle University, P.O. Box 231/1632, Mekelle, Ethiopia; ^2^Tigrai Biotechnology Center Pvt. Ltd. Co., Mekelle, Ethiopia; ^3^Mekhoni Agricultural Research Center, P.O. Box 71, Mekhoni, Ethiopia

## Abstract

This study aimed to describe the gross phytochemical constituents of *Aloe elegans* Todaro gel and evaluate the characteristics and quality of lab-made hair washing formulations prepared from the gel to show its potential in formulating hair washing shampoos. *A. elegans* gel mass was prepared from mature, healthy leaves collected from natural stand. Samples of 100% methanol extract of the gel were subjected to standard phytochemical screening and gas chromatography-mass spectroscopy (GC-MS) analysis. Five hair washing formulations (Fs) were, likewise, prepared by mixing 4.0–10.0 mL of gel with one (0.05 mL) to two (0.10 mL) drops of six synthetic and natural ingredients, namely, coconut oil, jojoba oil, olive oil, pure glycerin oil, lemon juice, and vitamin E. The gel to the total ingredient ratios (v/v) of the five formulations were 93 : 7 (F_1_), 94.5 : 5.5 (F_2_), 96.4 : 3.6 (F_3_), and 96.6 : 3.4 (F_4_ and F_5_). The formulations were evaluated using sensory inspection and common physicochemical methods. The phytochemical screening and GC-MS analysis revealed that *A. elegans* gel is the source of important chemical constituents used in the formulation of shampoos and similar products including saponins, capric acid, lauric acid, myristic acid, palmitic acid, linoleic acid, stearic acid, and phytol. Lab-made *A. elegans* hair washing formulations, especially those with 96.4–96.6% gel, were found to have similar characteristics and qualities with a common marketed shampoo. All the formulations were turbid with characteristic odor as the marketed shampoo. The pH values of the hair washing formulations (6.4–4.6) were comparable to those of the marketed shampoo (6.7). Formulations with higher proportion of gel had better foam stability, higher solid content (26–29%), higher surface tension (33–38 dynes/cm), shorter wetting time (150–160 sec), equivalent viscosities (26.45–26.73 poise), and conditioning performance than the marketed shampoo. These findings demonstrate that *A. elegans* gel mass can be used in the formulation of good-quality hair washing shampoos. We recommend future studies that aim to develop the phytochemical profile of the plant and a refined protocol of hair washing shampoo formulation.

## 1. Introduction

The genus *Aloe* L. (Aloaceae) comprises more than 500 species of succulent flowering plants. Aloes are native to many parts of Africa and Madagascar, the Mediterranean, Arabian Peninsula, South and Central America, the Rio Grande Valley of South Texas and Florida, Southern California and Mexico, the Pacific Rim Countries, India, the Caribbean, and Australia [1, 2]. They thrive in a range of latitudinal and altitudinal zones and diversity of habitats such as forests, wooded-grasslands, woodlands, rocky expanses, mountain tops and cliffs, beaches, waterfalls, and many other ecological zones with extreme environmental and soil conditions [3]. Fifty (50) species of *Aloe* are known and described in the flora of Ethiopia and Eritrea so far; 31 of them are endemic and restricted to very small areas [3, 4].

The Tigrai floristic region of the flora of Ethiopian and Eritrea is home to many *Aloe* species including *A. adigratana* Reynolds, *A. camperi* Schweinfurth, *A. elegans* Todaro, *A. macrocarpa* Todaro, *A. monticola* Reynolds, *A. percrassa* Todaro, *A. steudneri* Schweinfurth, and *A. trichosantha* Berger. *A. elegans* grows in a range of habitats in Tigrai, Wollo, Gojjam, and Shewa floristic regions of Ethiopia and Eritrea from 1,500 to 2,400 meters. The plant is the second or third most abundant *Aloe* species in Tigrai. It flowers from September to December and occasionally from March to May [3]. It is planted as fence of farm plots, backyards, homesteads, churchyards, forest enclosures, and footpaths in Tigrai. The conservation and sustainable use of this plant depends on the knowledge of its use and potential. The plant is included in the International Union for Conservation of Nature (IUCN) List of Threatened Species since 2013 [5].

Aloes have been all-purpose plants over many millennia across all civilizations. They are endowed with a range of chemical constituents that can be used in preparing beauty and cosmetic, medicinal and pharmaceutical, personal care and toiletry products, and bittering agents in alcoholic drinks. They are also grown as ornamental plants [6, 7]. Furthermore, aloes have been used in preparing many traditional remedies for healing wounds, anesthetizing tissues, stopping fungal, viral, and bacterial growths, improving blood flow, and acting as anti-inflammatory, antiaging, and antiallergic agents [6, 8]. Many varieties of *A. vera* L. (*A. barbadensis*) and few other species *A. arborescens*, *A. sinkatana*, *A. ferox*, and *A. pulcherrima* are extensively studied and are known to be rich sources of essential oils, fatty acids, alkaloids, and phenolic compounds that have a range of therapeutic and health benefits to humans [9–14]. Extracts and isolates of *Aloe* species are known to exhibit antioxidant, anticancer, anti-inflammatory, and antimicrobial activities. Thus, their economic potentials and applications in cosmetic and personal care, nutriceutical, pharmaceutical, and food and beverage industries are increasing [10, 15–17]. However, there are very limited studies on the environmental and commercial potentials of the majority of the aloes.

Many researchers explored into the phytochemistry, biocidal activities, and pharmaceutical properties in some Ethiopian aloes including *A. adigratana*, *A. citrina*, *A. debrana*, *A. elegans*, *A. harlana*, *A. megalacantha*, *A. otallensis*, *A. percrassa*, *A. pulcherrima*, *A. rivae*, and *A. sinana* [18–30]. However, the studies on Ethiopian aloes, including *A. elegans*, were isolated endeavors without continuity. One study with whole tissues of *A. elegans* leaf revealed that it is a good source of phenols, flavonoids, tannins, terpenoids, saponins, and glycosides with antifungal and antibacterial activities [21]. Another study showed that the plant is a useful source of traditional remedies against abdominal pain, malaria, diabetics, impotence, and many other human ailments in Tigrai (Ethiopia) and Eritrea [31, 32]. Moreover, it is used in the preparation of traditional hair washing shampoos by local people in Eritrea [31]. The present study aimed to determine the phytochemistry of *A. elegans* gel and evaluate the characteristics of lab-made hair washing formulations of the gel to demonstrate its potential for making hair washing and conditioning shampoo.

## 2. Materials and Methods

### 2.1. Collection and Preparation of Plant Specimens

Healthy and mature leaves of *A. elegans* were collected from wild stand at north of Sele (at about 86 km on the Mekelle-Abbiyi Addi highway; latitude/longitude: 13.560/39.026; altitude: 1,694 m) on 21 December 2018. Collection of plant materials by natives (Ethiopians) for research is granted by Article 15, Clause 1, of the Access to Genetic Resources and Community Knowledge and Community Rights Proclamation of the Federal Democratic Republic of Ethiopia (No. 482/2006). Specimens of the plant were identified by the National Herbarium (ETH), the Department of Biology, Addis Ababa University (Ethiopia). The leaves were rinsed with running tap water to remove any dirt and soil. The leaf (i.e., outer green skin) and the gel (i.e., inner gelatinous mass) were separated by peeling the skin off with a scalpel knife. The mass of gel was dried in shade at room temperature for 18 days. The dried mass of gel was then pulverized into powder using an electrical grinder and stored in a sealed container until used for phytochemical study.

### 2.2. Phytochemistry of Gel Extracts

Powder of *A. elegans* gel was extracted by 100% methanol using the continuous hot percolation method in Soxhlet apparatus for 18 hours. The extract was concentrated in a rotary evaporator into brown liquid and kept at 4°C in a deep freezer. Samples of the extract were taken out and subjected to phytochemical screening using the standard tests for alkaloids (Wagner test) [33], anthraquinones (Borntrager's test) [34], flavonoids (lead acetate test) [35], saponins (Froth test) [36], tannins (ferric chloride test) [37], and terpenoids (Salkowski test) [36].

### 2.3. Gas Chromatography-Mass Spectrometry (GC-MS) Analysis

Likewise, samples of the gel extract were sent to JIJE LOBOGLASS Plc.—a certified analytical laboratory in Addis Ababa, Ethiopia—for gas chromatography-mass spectroscopy (GC-MS) analysis. The instrument control parameters of the GC-MS are given in Appendix. The GC-MS analysis was carried out to determine the essential oil and fatty acid methyl ester contents of the gel.

### 2.4. Preparation of Hair Washing Formulations from *A. elegans* Gel

Five lab-made hair washing formulations were prepared by mixing *A. elegans* gel mass with six natural and formulated ingredients. The ingredients were coconut oil, jojoba oil, lemon juice, olive oil, pure glycerin oil, and vitamin E (Table 1).

The gel masses were mixed with the ingredients according to the ratio indicated in Table 2 to prepare enough volume hair washing formulations [38–40]. Then, mixtures were homogenized one by one with magnetic stirrer at 400 rpm for 30 min at 30°C, and white smooth formulations were obtained. Finally, the formulations were transferred into labeled collapsible plastic tubes and kept at room temperature for physicochemical evaluation.

### 2.5. Evaluation of the Characteristics of the Hair Washing Formulations

The formulations were evaluated via sensory inspection and physical assessment methods based on procedures developed and used by many researchers against a marketed shampoo (product name: Aloe Vera Hair Shampoo; producer: Perfect Cosmetics, UAE; size: 5.5 mL; description: natural blend of *Aloe vera* extract and moisturizers; manufactured: 02/2018; expiry: 02/2021) [38–45].

#### 2.5.1. Sensory Inspection

The physical appearance of the lab-made formulations was evaluated based their color, clarity, odor, consistency, and spreadability. The visual inspection of each formulation was carried out by three randomly selected volunteering students at room temperature.

#### 2.5.2. Determination of pH

A 10% (v/v) solution was prepared from each hair washing formulation using sterile distilled water. The pH of each solution was determined at room temperature (25°C) and recorded.

#### 2.5.3. Determination of Solids Contents

Four (4) grams of hair washing formulation was placed onto a clean, dry evaporating dish. The evaporating dish holding the shampoo was weighed using electronic balance, and the total weight was recorded as W_1_. Then, the evaporating dish was placed on the hot plate at 50°C and was kept until the liquid content was completely evaporated. Finally, the cooled evaporating dish holding the solid content was weighed and recorded as *W*_2_, and the percentage (%) of the solid content was calculated as [(*W*_1_–*W*_2_) ÷ *W*_1_] × 100.

#### 2.5.4. Measurement of Surface Tension

Surface tension measurements were carried out using a 10% (v/v) solution of hair washing formulation prepared with sterile distilled water at 25°C. The surface tension of each solution was measured by the stalagmometric method. Thus, the stalagmometer was thoroughly cleaned using chromic acid and sterile distilled water to remove any traces of greases and lubricants because they greatly affect surface tension. The surface tension was calculated using this formula *R*^2^ = [((*W*_3_–*W*_1_) × *N*_1_) ÷ ((*W*_2_–*W*_1_) × *N*_2_)] × *R*_1_, where *W*_1_ is the weight of empty beaker, *W*_2_ is the weight of beaker with distilled water, *W*_3_ is the weight of beaker with formulation solution, *N*_1_ is the number of drops of distilled water, *N*_2_ is the number of drops of formulation solution, *R*_1_ is the surface tension of distilled water at 25°C, and *R*^2^ is the surface tension of shampoo solution at 25°C [41].

#### 2.5.5. Dirt Dispersion

Two drops (0.10 mL) formulation was added into a 100 mL test tube containing 10 mL sterile distilled water. Then, one drop (0.05 mL) of India ink was added to the test tube, stoppered, and shaken 10 times. The amount of ink in the foam was estimated as none, light, moderate, and heavy by three randomly selected volunteering students.

#### 2.5.6. Rheological (Viscosity) Evaluations

The viscosities of the hair washing formulations were determined using the Brookfield Viscometer (Model DV-l Plus, LV, USA) set at different spindle speeds ranging from 0.3 to 10 rpm. Viscosity measurements were carried out using spindle T95 by maintaining the temperature at 25°C and the sizes of the containers holding formulation samples constant.

#### 2.5.7. Foaming Ability and Foam Stability

The foaming ability of the formulations was determined using the cylinder shake method at 25°C. Fifty (50) mL of the 1% (v/v) solution of hair washing formulation was put into a 250 mL graduated cylinder. The cylinder was covered by hand, shaken 10 times, and left to stand for 1 min in a test tube rack. The volume of the foam was recorded at the end of 1 min standing. This represented the foaming ability. The foaming stability was, likewise, determined by measuring the volume of the foam at 1, 2, 3, and 4 min after shaking and observing the decrement in foam volume [44].

#### 2.5.8. Wetting Time Test

The wetting times of the hair washing formulations were determined using the canvas disc method with some modifications [38–40, 45]. One (1) inch diameter discs, weighing 0.45 grams, were cut out from a smooth garment (velvet). Also, 400 mL of 1% (v/v) solutions were prepared in a 500 mL graduated cylinder from all the formulations. Wetting time of each formulation was tested by floating a canvas disc on the graduated cylinder holding the 400 mL solution and recording the time required for the disk to start sinking using a stopwatch. The time required for the disc to start sinking was recorded as wetting time.

#### 2.5.9. Evaluation of Conditioning Performance

Conditioning performances of the formulations were evaluated using the procedure developed by Boonme et al. [43] with some modifications. Ethiopian male hair cut was collected from barber shop and divided into 5 g mass. One 5 g mass served as control and another 5 g mass was washed with each formulation. Washing solution was prepared by mixing 1 mL of formulation and 50 mL of distilled water in a conical flask. Then, the 5 g hair mass was put into the flask, shaken for 2 min, rinsed with 100 mL distilled water, placed in plastic sheet, and allowed to dry at room temperature. The control hair mass was washed with distilled water only. Finally, the smoothness and softness (i.e., conditioning performance) of the hair mass was estimated by blind touch test methods involving three randomly selected volunteering students. The students were blind folded and asked to touch (feel) the hair mass for its smoothness and softness, and rate it as poor, satisfactory, good, and excellent. They also visually inspected the hair mass for its glowing appearance and silkiness.

## 3. Results and Discussion

### 3.1. Phytochemistry of *A. elegans* Gel

Like many *Aloe* species, *A. elegans* is the source of many useful phytochemicals. The present phytochemical screening using methanol gel extracts of the species yielded positive results for anthraquinones, flavonoids, saponins, and tannins (Table 3). Another study reported the presence of terpenoids by using ethyl acetate extract [21].

**Figure 1:**
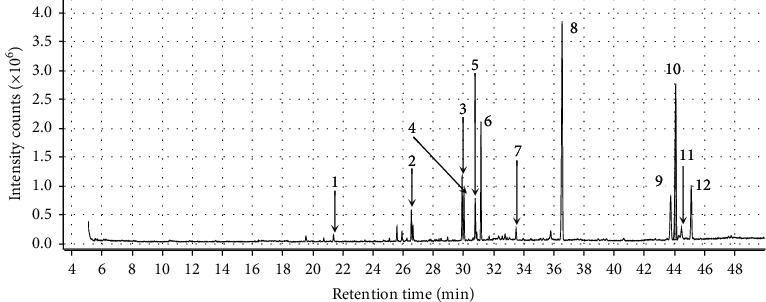
GC-MS analysis of *A. elegans* leaf gel extract.

GC-MS analysis of gel extract of *A. elegans* resulted in 12 compounds (Table 4, Figure 1). The compounds or their derivatives are used in formulating beauty and personal care products. Decanoic (capric) acid (**1**), dodecanoic (lauric) acid (**2**), hexadecanoic (palmitic) acid (**8**), (Z,Z)-9,12-octadecadienoic (linoleic) acid (**9**), and phytol (**11**) are used in preparing personal care products such as soaps and detergents. Moreover, decanoic (capric) acid (**1**), tetradecanoic (myristic) acid (**6**), hexadecanoic (palmitic) acid (**8**), (Z,Z)-9,12-octadecadienoic (linoleic) acid (**9**), and phytol (**11**) are used to formulate cosmetics and beauty products. Phytol (**11**) and octadecanoic (stearic) acid (**12**) are also important components in producing commercial shampoos and shaving creams. Furthermore, whereas compound **9** is important source of surfactants, compound **12** is used in saponification [46]. Similar fatty acids and essential oils were found in *A. adigratana* Reynolds [38].

### 3.2. Evaluation of Hair Washing Formulations of *A. elegans* Gel

#### 3.2.1. Sensory Assessment

Cosmetic products including hair washing shampoos have attractive appearance to the sensory observer. Sensory observation and simple measurements showed that the formulations were white and turbid with characteristically good odor without major difference from the commercial shampoo (Table 5). The white color of the formulations was due to the absence of the coloring agent. The color, turbidity, and odor of the shampoos did not change with increasing the proportion of the gel.

Formulations 1–4 containing 4–10 mL gel are thinner. Slightly thick formulation, such as the marketed shampoo, was produced with 10 mL of gel and 2 drops coconut oil in F_5_. Likewise, formulations with higher gel volume (8–10 mL) showed best consistency as the marketed shampoo. Varying the proportion of the gel did not bring about significant change in pH of the formulations—falling within the pH range of many marketed shampoos [38–40, 47].

#### 3.2.2. Quality Characteristics

Solid content, foam stability, dirt dispersion, surface tension, wetting time, and conditioning performance are the principal parameters used in the qualitative evaluation of shampoos. The physical characteristics of the hair washing laboratory formulations are summarized in Table 6.

*(1). Solid Content*. The solid contents of our formulations ranged from 23% to 29% (Table 6). Quality shampoos are preferred to have the solid content of 20%–30%. Shampoos with lower solid content are thin and watery while those with higher solid content are thick and greasy. Whereas thin shampoos drain off the hair quickly, the thick ones are difficult to work with [39, 41, 48, 49]. Increasing the proportion of the gel from 2 mL to 10 mL consistently led to the raising of the solid contents of the formulations. Thus, the solid content of *A. elegans*-based hair washing formulations can be easily decided by fixing the proportion of the gel [38].

*(2). Foam Ability and Stability*. Good shampoos have bigger and stable foams upon shaking. Foam volume and stability are principal quality parameters of shampoos [50]. All our formulations were very good in terms of volume and stability similar to the marketed *A. vera* shampoo. All the formulations were compact, uniform, and stable maintaining their volumes for more than four minutes. Similar findings were reported with lab-made *A. adigratana* formulations [38].

*(3). Dirt Dispersion*. Shampoos that concentrate dirt or stain in their foams are regarded as low quality [39, 40, 51]. But good shampoos and detergents concentrate the dirt in the water. Since the dirt is often water insoluble, it is removed by the help of surfactants present in the shampoos and detergents. The present study showed that all the formulations yielded clean foams with no dirt like the marketed shampoo (Table 6). These imply that the linoleic acid (Compound **9**) present in the leaf gel of the species is an important source of surfactants. Similar observations were reported elsewhere [38, 48, 52].

*(4). Surface Tension*. Good shampoos and detergents reduce the surface tension of pure water from 72 dynes/cm to below 40 dynes/cm at 25°C [53] to improve detergency [54]. Our study resulted in *A. elegans* formulations with surface tension ranging from 33 (10 mL gel) to 38 dynes/cm (4 mL gel) at 25°C (Table 6). Other researchers also formulated herbal shampoos with surface tension between 30 and 40 dynes/cm [38, 49, 50]. The decrease of the surface tension of the formulations with increasing the proportion of gel might be accounted for the amount of surfactants (linoleic acid) in the gel. Increasing the proportion of the *A. elegans* gel resulted in formulations with surface tension comparable to that of the marketed shampoo [38].

*(5). Wetting Ability*. Shampoos with high concentration of surfactants have better wetting abilities. Canvas disc tests resulted that our *A. elegans* hair washing formulations have lower wetting time (142–160 sec) compared to the market *A. vera* shampoo (185 sec) (Table 6). Higher concentration of detergents causes lower wetting time [38, 42].

*(6). Conditioning Performance*. The conditioning performances of shampoos are largely affected by their chemical properties. They are, therefore, formulated by enriching them with conditioning agents. The agents deposit, adhere, or adsorb onto proteins of hair and improve its manageability. They also reduce hair static and make it soft and smooth [42]. Samples of Ethiopian cut hair washed with the formulations became smooth and soft as compared to that washed with pure water. Thus, all the formulations demonstrated that good conditioning performance rendered the hair samples glowing, soft, silky, and manageable. The conditioning performance of the formulations may be accounted to the capric (**1**), lauric (**2**), myristic (**6**), palmitic (**8**), and stearic (**12**) acids detected in the gel. Fatty acids with 8–18 carbons are used in formulating shampoos and conditioners [46, 55].

*(7). Viscosity*. Viscosity affects the spreadability and consistency of shampoos. Good shampoos spread easily upon application and remain consistent until use [41]. The viscosities of the formulations of the present study ranged from 13.56 (4 mL gel) to 26.73 poise (10 mL gel). Viscosities of the formulations prepared using 6–10 mL of gel are close to that of the marketed shampoo (26.45 poise) (Table 7). The viscosities of the formulations were increased by increasing the amount of the gel. The moisture contents of the formulations were also ranged between 95% and 96%. Therefore, *A. elegans* formulations with higher proportion of gel were found to have comparable viscosities and other properties with the marketed shampoo [38–40].

## 4. Conclusion

The phytochemical screening and GC-MS analysis of methanol extract revealed that *A. elegans* gel is a good source of key chemical constituents used in the formulation of beauty, cosmetics, detergent, and personal care products. Likewise, the lab-made hair washing formulations prepared and evaluated in this study exhibited desirable properties recommended for similar products. Evaluation through sensory observation and physicochemical tests revealed that *A. elegans* gel demonstrated good potential for formulating hair shampoos. Thus, future studies may aim at establishing the complete phytochemical profile of the plant and developing refined shampoo-formulation protocol. Further work on this threatened species would encourage efforts towards its conservation and sustainable use.

## Figures and Tables

**Table 1:** Ingredients used in preparing lab-made hair washing formulations from *A. elegans* gel.

Generic name	Manufacturer	Biological applications
*A. elegans* gel	—	Repairs, strengthens, hydrates, and softens hair; makes hair look and feel healthier; heals wounds; acts as natural surfactant
Coconut oil	C.B.C., Malaysia	Prevents protein loss in hair; moisturizes skin; acts as a natural sunscreen
Jojoba oil	ORS, USA	Moisturizes and gives hair shining look
Lemon juice	Fresh lab extract	Acts as natural antioxidant, chelating, and antidandruff agent; maintains the pH of the acidic formulation
Olive oil	Salamati, Spain	Moisturizes hair; minimizes scalp irritation; reduces dandruff
Pure glycerin oil	LFRESSH-Eurogulf, UAE	Hydrates skin; enhances cell maturation; removes dandruff
Vitamin E	Fruit of the Earth, USA	Supports scalp; gives hair strong base to grow; reduces oxidative stress; preserves protective lipid layer

**Table 2:** Preparation of hair washing formulations from *A. elegans* gel.

Ingredients	UoM^*∗*^	Formulations									
		F_1_		F_2_		F_3_		F_4_		F_5_	
		Vol.	% v/v	Vol.	% v/v	Vol.	% v/v	Vol.	% v/v	Vol.	% v/v
*A. elegans* gel	mL	4.0	93.0	6	94.5	8	96.4	10	96.6	10	96.6
Coconut oil	gtt.	1	1.16	1	0.79	1	0.60	1	0.48	2	0.97
Olive oil	gtt.	1	1.16	2	1.57	1	0.60	2	0.97	1	0.48
Jojoba oil	gtt.	1	1.16	1	0.79	1	0.60	1	0.48	1	0.48
Glycerin oil	gtt.	1	1.16	1	0.79	1	0.60	1	0.48	1	0.48
Vitamin E	gtt.	1	1.16	1	0.79	1	0.60	1	0.48	1	0.48
Lemon juice	gtt.	1	1.16	1	0.79	1	0.60	1	0.48	1	0.48

^*∗*^UoM, unit of measurement; mL: milliliter; gtt.: drop (0.05 mL).

**Table 3:** Phytochemical screening of *A. elegans* leaf gel.

Phytochemicals	Tests	Inspection	Results	Reference
Alkaloids	Wagner test	Brownish-red precipitate	−	[33]
Anthraquinones	Borntrager's test	Pink, red	+	[34]
Flavonoids	Lead acetate test	Yellow precipitate	+	[35]
Saponins	Froth test	Foam	+	[36]
Tannins	Ferric chloride test	Dark-green	+	[37]
Terpenoids	Salkowski test	Reddish-brown	−	[36]

“+” sign indicates the presence, and “–” sign indicates absence of the chemical constituents.

**Table 4:** Chemical composition of A. *elegans* leaf gel extract.

SN	Name	Formula	Area	RT	% area
1	Decanoic (capric) acid, methyl ester	C_11_H_22_O_2_	408,497	21.37	0.69
2	Dodecanoic (lauric) acid, methyl ester	C_13_H_26_O_2_	1,516,579	26.55	2.55
3	Ar-tumerone	C_15_H_20_O	3,401,566	29.92	5.72
4	Tumerone	C_15_H_22_O	2,691,776	30.04	4.53
5	Curlone	C_15_H_22_O	2,261,927	30.79	3.81
6	Tetradecanoic (myristic) acid, methyl ester	C_15_H_30_O_2_	5,658,349	31.16	9.52
7	9-Methyltetradecanoic (9-methylmyristic) acid, methyl ester	C_16_H_32_O_2_	661,028	33.50	1.11
8	Hexadecanoic (palmitic) acid, methyl ester	C_17_H_34_O_2_	18,109,462	36.55	30.47
9	(Z,Z)-9,12-octadecadienoic (linoleic) acid, methyl ester	C_19_H_34_O_2_	4,180,215	43.76	7.03
10	(E)-9-octadecadienoic (elaidic) acid, methyl ester	C_19_H_36_O_2_	14,946,083	44.09	25.14
11	Phytol	C_20_H_40_O	1,229,919	44.48	2.07
12	Octadecanoic (stearic) acid, methyl ester	C_19_H_38_O_2_	4,376,317	45.12	7.36

**Table 5:** Physical inspection of hair washing formulations of *A. elegans* gel.

Formulations	Color	Clarity	Odor	Consistency	Spreadability	pH	Temperature (°C)
F_1_ (4 mL)	White	Turbid	Characteristic	Thin	Good	6.5	25
F_2_ (6 mL)	White	Turbid	Characteristic	Thin	Good	6.6	25
F_3_ (8 mL)	White	Turbid	Characteristic	Thin	Best	6.6	25
F_4_ (10 mL)	White	Turbid	Characteristic	Thin	Best	6.5	25
F_5_ (10 mL)	White	Turbid	Characteristic	Slightly thick	Best	6.4	25
Marketed	Green	Turbid	Characteristic	Slightly thick	Best	6.7	25

**Table 6:** Evaluations of *A. elegans* hair washing formulations.

Formulation	Solid content (%)	Foam stability	Dirt in the foam	Surface tension (dynes/cm)	Wetting time (sec.)	Conditioning performance	Temp. (°C)
F_1_ (4 mL)	23	Very good	Not detected	38	142	Good	25
F_2_ (6 mL)	24	Very good	Not detected	37	150	Good	25
F_3_ (8 mL)	26	Very good	Not detected	36	152	Good	25
F_4_ (10 mL)	28	Very good	Not detected	34	153	Good	25
F_5_ (10 mL)	29	Very good	Not detected	33	160	Good	25
Marketed	26	Very good	Not detected	32	185	Good	25

**Table 7:** Viscosities of *A. elegans* gel formulations.

Formulations	Viscosity (poise)	Speed (rpm)	% FSR	Shear stress	Stress rate	Temperature (°C)
F_1_ (4 mL)	13.56	60	37.89	112.87	899.99	25
F_2_ (6 mL)	26.47	60	66.67	168.38	899.99	25
F_3_ (8 mL)	26.66	60	69.52	169.94	899.99	25
F_4_ (10 mL)	26.73	60	69.11	169.72	899.99	25
F_5_ (10 mL)	26.45	60	68.78	169.11	899.99	25
Marketed	26.92	60	69.98	169.91	899.99	25

## Data Availability

The datasets used and/or analyzed during the current study are available from the corresponding author upon reasonable request.

## Appendix

### Instrument Control Parameters of GC-MS

D:\MassHunter\GCMS\1\methods\Fatty Acid_*A. elgans*_DB5MS 10.M Wed Jul 17 11 : 39 : 33 2019; control information: sample inlet, GC; injection source, GC ALS; injection location, front, and mass spectrometer, enabled. GC: oven temperature; set point On (initial) 60°C; hold time, 0 min and post run, 50°C. Program: #1 Rate 3°C/min; #1 Value 110°C; #1 Hold Time 0 min; #2 Rate 10°C/min; #2 Value 140°C; #2 Hold Time 1 min; #3 Rate 5°C/min; #3 Value 195°C; #3 Hold Time 10 min; #4 Rate 5°C/min; #4 Value 225°C; #4 Hold Time 6 min; #5 Rate 20°C/min; #5 Value 250°C and #5 Hold Time 4 min.

## References

[1] IASC (2002). *Aloe Vera: A Long, Illustrious History Dating From Biblical Times*.

[2] Dwivedi N., Indiradevi A., Asha K., Asokan N., Suma A. (2014). A protocol for micropropagation of *Aloe vera* L. (Indian Aloe)–a miracle plant. *Research in Biotech*.

[3] Demissew S., Nordal I. (2010). *Lilies and Aloes of Ethiopia and Eritrea*.

[4] Demissew S., Friis I., Feye T. A. (2011). Four new species of Aloe (Aloaceae) from Ethiopia, with notes on the ethics of describing new taxa from foreign countries. *Kew Bulletin*.

[5] Weber O. (2013). *“Aloe elegans*.

[6] Sahu P., Giri D., Singh R. (2013). Therapeutic and medicinal uses of *Aloe vera*: a review. *Pharmacology & Pharmacy*.

[7] Adelberg J., Naylor-Adelberg J. (2012). Effects of cytokinin on multiplication and rooting of Aloe barbadensis during micropropagation on agar and liquid media. *Journal of Medicinally Active Plants*.

[8] Yao H., Chen Y., Huang L., Chen W., Lin X. (2009). Promotion proliferation effect of polysaccharide from Aloe barbadensis Miller on human fibroblasts in vitro. *International Journal of Biological Macromolecules*.

[9] Periasamy G., Kassa S., Sintayehu B., Mebrahtom G., Geremedhin G., Karim A. (2014). Cosmetic use of Aloe vera–a review. *World Journal of Pharmacy and Parmaceutical Sciences*.

[10] Adesuyi A. O., Awosanya O. A., Adaramola F. B., Omeonu A. I. (2012). Nutritional and phytochemical screening of Aloe barbadensis. *Current Research of Journal Biological Sciences*.

[11] Rodríguez E. R., Martín J. D., Romero C. D. (2010). Aloe vera as a functional ingredient in foods. *Critical Reviews in Food Science and Nutrition*.

[12] Elhassan G. O. M., Adhikari A., Yousuf S. (2012). Phytochemistry and antiglycation activity of Aloe sinkatana reynolds. *Phytochemistry Letters*.

[13] Chen W., Wyk B. V., Vermaak I., Viljoen A. M. (2012). Cape aloes-A review of the phytochemistry, pharmacology and commercialisation of Aloe ferox. *Phytochem. Lett.*.

[14] Park M. K., Park J. H., Kim N. Y. (1998). Analysis of 13 phenolic compounds in Aloe species by high performance liquid chromatography. *Phytochemical Analysis*.

[15] Sánchez-Machado D. I., Lopez-Cervantes J., Sendon R., Sanches-Silva A. (2017). Aloe vera : ancient knowledge with new frontiers. *Trends in Food Science & Technology*.

[16] Maan A. A., Nazir A., Kashif M. (2018). The therapeutic properties and applications of Aloe vera: a review. *Journal of Herbal Medicine*.

[17] Salehi B., Albayrak S., Antolak H. (2018). Aloe genus plants: from farm to food applications and phytopharmacotherapy. *International Journal of Molecular Sciences*.

[18] Lidia L. B., Sibhatu D. B., Seid M. (2019). Comparative antimicrobial activities of the gel, leaf and anthraquinone fractionates of four Aloe species (Aloe camperi, Aloe elegans, Aloe eumassawana and Aloe schoelleri). *Advances in Microbiology*.

[19] Brhane G. H., Gopalakrishnan V. K., Hagos Z., Hiruy M., Devaki K., Chaithanya K. K. (2018). Phytochemical screening and in vitro antioxidant activities of ethanolic gel extract of Aloe adigratana Reynolds. *Journal of Pharmacy Research*.

[20] Tsegay M., Tewabe Y., Bisrat D., Asres K. (2018). In vivo anti-inflammatory activity of two anthrones from the leaves of Aloe adigratana Reynolds and Aloe elegans Todaro. *Ethiopian Pharmaceutical Journal*.

[21] Habtemariam M., Medhanie G. (2017). Screening of biologically active constituents from leaves of Aloe elegans and their antimicrobial activities against clinical pathogens. *African Journal of Microbiology Research*.

[22] Girma B., Bisrat D., Asres K. (2015). Antimalarial evaluation of the leaf latex of Aloe citrina and its major constituents. *Ancient Science of Life*.

[23] Gebremedhin G., Bisrat D., Asres K. (2014). Isolation, characterization, and in vivo antimicrobial evaluation of anthrones from leaf latex of Aloe percrassa Todaro. *Journal of Natural Remedies*.

[24] Gemechu W., Bisrat D., Asres K. (2014). Antimalarial anthrone and chromone from the leaf latex of. *Ethiopian Pharmaecutical Journal*.

[25] Minale G., Bisrat D., Asres K., Mazumder A. (2014). In vitro antimicrobial activities of anthrones from the leaf latex of Aloe sinana Reynolds. *International Journal of Green Pharmacy*.

[26] Suleyman A., Gnanasekaran N., Daniel S. (2014). Amelioration of streptozotocin-induced hyper-glycemia and dyslipidemia through Aloe debrana. *International Journal of Pharmacy and Pharmaceutical Sciences*.

[27] Asamenew G., Bisrat D., Mazumder A., Asres K. (2011). In vitro antimicrobial and antioxidant activities of anthrone and chromone from the latex of aloe harlana Reynolds. *Phytotherapy Research*.

[28] Paulos B., Bisrat D., Gedif T., Asres K. (2011). Antimalarial and antioxidant activities of the leaf exudates and a naphthalene derivative from Aloe otallensis Baker. *Ethiopian Pharmaceutical Journal*.

[29] Deressa T., Mekonnen Y., Animut A. (2010). In vivo antimalarial activities of Clerodendrom myricoides, Dodonaea angustifolia and Aloe debrana against Plasmodium berghei. *Ethiopian Journal of Health and Development*.

[30] Dagne E., Alemu M. (1991). Constituents of the leaves of four Aloe species from Ethiopia. *Bulletin of the Chemical Society of Ethiopia*.

[31] Yemane B., Medhanie G. (2016). Ethnobotanical study of medicinal plants in sub-zoba debarwa, zoba debub, Eritrea. *Eritrean Journal of Science and Engineering*.

[32] Beyene T. (2015). Ethnobotany of medicinal plants in erob and gulomekada districts, Eastern zone of Tigrai Region, Ethiopia.

[33] Parekh J., Karathia N., Chanda S. (2006). Evaluation of antibacterial activity and phytochemical analysis of Bauhinia variegata L. bark. *African Journal of Biomedical Research*.

[34] Kebede T., Kibret F., Fikre M., Milkyas E. (2015). Phytochemical screening and characterization of olean-18-ene type triterpeniod from the roots of. *Science, Technology and Arts Research Journal*.

[35] Bhandary S., Kumari S., Bhat V., Sherly S., Bekal M. P. (2012). Preliminary phytochemical screening of various extracts of Punica granatum peel, whole fruit and seeds. *Journal of Health Sciences*.

[36] Abebayehu A., Mammo F., Kibret B. (2016). Isolation and characterization of terpenes from leaves of Croton macrostachyus (Bissana). *Journal of Medicinal Plants Research*.

[37] Sofowora A. (1982). *Medicinal Plants and Traditional Medicine in Africa*.

[38] Sbhatu D. B., Berhe G. G., Hndeya A. G. (2020). Formulation and physicochemical evaluation of lab-based Aloe adigratana Reynolds shampoos. *International Journal of Analytical Chemistry*.

[39] Al Badi K., Khan S. A. (2014). Formulation, evaluation and comparison of the herbal shampoo with the commercial shampoos. *Beni-Suef University Journal of Basic and Applied Sciences*.

[40] Pounikar Y., Jain P., Khurana N., Omray L. K., Patil S., Gajbhiye A. (2012). Formulation and characterization of Aloe vera cosmetic herbal hydrogel. *International Journal of Pharmacy and Pharmaceutical Sciences*.

[41] AlQuadeib B. T., Eltahir E. K. D., Banafa R. A., Al-Hadhairi L. A. (2018). Pharmaceutical evaluation of different shampoo brands in local Saudi market. *Saudi Pharmaceutical Journal*.

[42] Shinde P. R., Tatiya A. U., Surana S. J. (2013). Formulation, development and evaluation of herbal antidandruff shampoo. *International Journal of Research in Cosmetic Science*.

[43] Boonme P., Pakpayat N., Yotmanee K., Kunlawijitrungsee S., Maneenuan D. (2011). Evaluation of shampoos containing silicone quaternary microemulsions. *Journal of Applied Pharmaceutical Science*.

[44] Manikar A. R., Jolly C. I. (2000). Evaluation of commercial herbal shampoos. *International Journal of Cosmetic Science*.

[45] Klein K. (2004). Evaluation of shampoo foam. *Cosmetics and Toiletry Management*.

[46] NIH (US National Library of Medicine) (2020). National center for biotechnology information, (National Institute of Health). http://pubchem.ncbi.nlm.nih.gov.

[47] Tarun J., Susan J., Suria J., Susan V. J., Criton S. (2014). Evaluation of pH of bathing soaps and shampoos for skin and hair. *Indian Journal of Dermatology*.

[48] Bakr R. O., Amer R. I., Fayed M. A. A., Ragab T. I. M. (2019). A completely polyherbal conditioning and antioxidant shampoos: a phytochemical study pharmaceutical evaluation. *Journal of Pharmacy and Bioallied Sciences*.

[49] Vijayalakshmi A., Sangeetha S., Ranjith N. (2018). Formulation and evaluation of herbal shampoo. *Asian Journal of Pharmaceutical and Clinical Research*.

[50] Deeksha R., Malviya P., Kumar S. (2014). Evaluation of marketed shampoo (synthetic and natural) for their hair cleansing, dirt dispersion, wetting time, solid content and foaming capacity properties. *Global Journal of Pharmacology*.

[51] Saad A. H., Kadhim R. B. (2011). Formulation and evaluation of herbal shampoo from Ziziphus spina-christi leaves extract. *International Journal of Research in Ayurveda & Pharmacy*.

[52] Yang J., Sakamoto K., Lochhead R. Y., Maibach H. I., Yamashita Y. (2017). Hair care cosmetics. *Cosmetic Science and Technology: Theoretical Principles and Application*.

[53] Ireland S., Carlino K., Gould L. (2007). Shampoo after craniotomy: a pilot study. *Canadian Journal of Neuroscience*.

[54] Kumar A., Mali R. R. (2010). Evaluation of prepared shampoo formulations and compare formulated shampoo with marketed shampoos. *International Journal of Pharmaceutical Sciences Review and Research*.

[55] Fealy B. J., Reinbold A. I. (1995). Shampoo and conditioning composition. https://patents.google.com/patent/US5656257A/en.

